# Suitability of Highly Polymerised Polyaluminium Chlorides (PACls) in the Treatment of Mixture of Groundwater and Surface Water

**DOI:** 10.3390/molecules28020468

**Published:** 2023-01-04

**Authors:** Izabela Krupińska

**Affiliations:** Faculty of Civil Engineering, Architecture and Environmental Engineering, Institute of Environmental Engineering, University of Zielona Góra, 15 Prof. Z. Szafrana St, 65-516 Zielona Góra, Poland; i.krupinska@iis.uz.zgora.pl; Tel.: +48-68-3282637

**Keywords:** coagulation, prehydrolysed coagulants, polyaluminium chlorides, organic matter, iron

## Abstract

The aim of this study was to evaluate the effectiveness of the coagulation process using highly polymerised polyaluminium chlorides in reducing the level of pollution of water in a mixture of groundwater and surface water. The coagulants used were prehydrolysed polyaluminium chlorides with the trade names PAXHP908 and PAXXL1911 that had alkalinity 85% and different iron contents (<0.01% and 0.7%). The Al species distribution in the PACls (PAXXL1911 ad PAXHP908) samples were analysed by the Ferron complexation timed spectrophotometry. The content of polymer forms of aluminium (Al_b_) in the tested coagulants was 40%. The worse results in the removal of organic matter (TOC, DOC, UV_254_), iron, colour and turbidity in the coagulation process were produced by the PAXXL1911, possessing higher content of iron (0.7%). The lower usefulness of the PAXXL1911 was probably caused by the interaction of organic ligands present in the treated water and Fe(III) ions introduced into the water with the coagulant. The effectiveness of the coagulation process with the tested coagulants was also evaluated by measuring the electrokinetic potential ζ, which determines the stability of the colloidal system.

## 1. Introduction

Groundwater and surface waters constitute the principal source of raw water for drinking water production. There are serious problems regarding the water supplies in towns with high density of population, because the quantity and quality of groundwater are not sufficient to ensure the total potable water demands. It is necessary to use surface water or a mixture of groundwater and surface water with increased organic pollutants content to produce potable water. The problem of how to increase the efficiency of coagulation for the removal of organic pollutants from surface water and groundwater, which are precursors of disinfection by-products and also contributors to the biological stability of the water is still raising interest. Some investigators place great emphasis on the coagulants which destabilise colloids and neutralise the charge of organic anions, others point to the presence of the polymeric forms of the hydrolysis products, which induce bridging coagulation. Regardless of the mechanism, experience has shown that non-prehydrolysed coagulants, e.g., aluminium sulphate (VI), provide the best organic matter removal in an acidic environment, which is generally obtained by acidifying the raw water or applying increased doses of hydrolysing coagulants. Unfortunately, both of these methods have the disadvantage of increasing undesirably the corrosive tendency of water. To minimise this adverse effect it is advisable to replace aluminium sulphate (VI) with prehydrolysed coagulants, which contain polymerised products of the prehydrolysis of aluminium and have a high positive charge. There are various types of prepolymerised coagulants, such as polyaluminium chloride (PAC), polysilicic acid (PSi) and polyferric sulfate (PFS). Among them, polyaluminium chloride is the most widely used in water purification due to its fast flocculation speed and large floc size [1,2,3,4,5,6,7]. In water treatment plants, in order to improve the efficiency of coagulation process, the use of prehydrolysed coagulants in place of aluminium sulphate (VI) is a common practice. Polyaluminium chlorides, as trade products, are characterised by alkalinity ranging from 15 to 90%. Coagulants of low alkalinity are characterised by alkalinity below 40%, average alkalinity (from 40 to 70%) and high alkalinity (from 80 to 85%).

Prehydrolysed reagents based on polymerised forms of aluminium generally are more effective in removing natural organic matter compared to non-prehydrolysed coagulants e.g., aluminium sulphate (VI) [8,9,10,11]. Polyaluminium chlorides represented by the general formula Al_n_(OH)_m_Cl_3n–m_ are primarily used. The alkalinity of polyaluminium chloride is determined by the quotient of the number of moles OH^−^ to Al^3+^ in a coagulant, referred to as the alkalinity ratio (r), which is approached as a coagulant polymerisation degree measure. The “Al_13_” polymer (Al_13_O_4_(OH)_24_^7+^) is the most stable and efficient in colloid destabilisation of all aluminium polymers and its amount increases along with the alkalinity ratio (r) increase to 2.1. The degree of polymerisation can be determined by means of ferron-timed spectroscopy method. The different species can be classified into three categories: the monomeric (Al_a_), polymerised (Al_b_) and precipitated/colloidal (Al_c_) species, in which the Al_b_ species are considered to be the most efficient species for DOC (dissolved organic carbon) removal [12]. These coagulant species (Al_b_) are considered to be the most efficient Al-species due to their larger size and higher positive charges [13,14,15,16,17,18,19,20,21].

The greater the degree of initial hydrolysis of coagulants (higher value of the alkalinity coefficient (r = [OH^−^]/[Al^3+^]), the lower is the aggressiveness of the acid carbonic, which for polyaluminium chloride explains reactions 1–4.

For r = 1 Al(OH)Cl_2_ + 2H_2_O → Al(OH)_3_ + 2HCl (1)

2HCl + Ca(HCO_3_)_2_ → CaCl_2_ + 2H_2_O + 2CO_2_ (2)

For r = 2 Al(OH)_2_Cl + H_2_O → Al(OH)_3_ + HCl (3)

HCl + 0.5Ca(HCO_3_)_2_ → 0.5CaCl_2_ + H_2_O + CO_2_ (4)

Increased efficacy of reagent containing prehydrolysis products implies the possibility of reduction of the required dose [22,23]. The polymeric structure of the hydrolysis products causes polyaluminium chlorides to show agglomerating properties, thus improving flocculation conditions. The effectiveness of coagulation to remove organic substances depends on several factors, including coagulant type and dose, mixing conditions, pH, temperature, particle and organic substances properties. Although the mechanism of coagulation with non-prehydrolysed and prehydrolysed aluminium salts is the same, the presence of polymeric forms of aluminium in polyaluminium chloride solutions makes them more stable in water, providing more effective removal of impurities and lower concentrations of aluminium remaining in the treated water. In spite of the development of new separation techniques for water treatment, the coagulation process will continue to be the most important water-treatment process for the removal of organic substances [24,25,26,27].

The goal of the present study was to evaluate the effectiveness of the coagulation process using highly polymerised polyaluminium chlorides with the same alkalinity (85%) but differing in iron content (0.7% and <0.01%) in reducing the level of pollution of water being a mixture of groundwater and surface water, with particular emphasis on the removal of organic substances and iron compounds. Apart from the typical indicators used to evaluate the content of organic matter, TOC and DOC, absorbance in the ultraviolet UV_254_ was also determined which indirectly determines the content of dissolved organic substances containing aromatic rings. Those are characterised by a high potential of forming oxidation or disinfection by-products.

## 2. Results and Discussion

**Figure 1:**
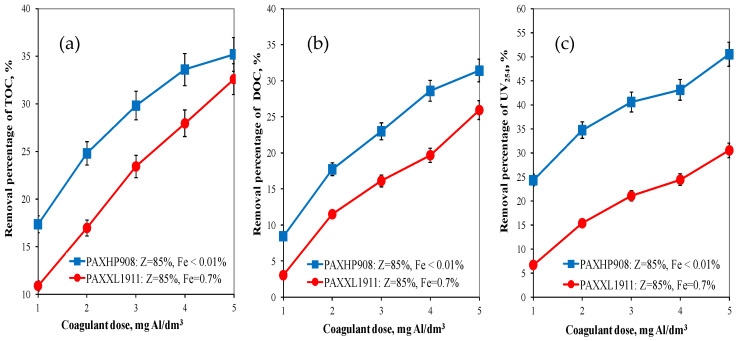
The effect of the type and dose of a coagulant on the efficiency of removing TOC (**a**), DOC (**b**) and UV_254_ (**c**).

On the basis of the obtained test results, the effectiveness of organic substances removal from water was determined by the coagulation method depending on the type and dose of the tested aluminium coagulant. Analysis of the results obtained showed that the effectiveness of removing organic pollution (TOC, DOC, UV_254_) increased as the doses of the coagulants tested increased. The higher efficiency of TOC (from 17.35 to 35.2%) and DOC (from 8.44 to 31.4%) removal from water was provided by the coagulant PAXHP908 with an alkalinity of 85%, no iron in its composition (Figure 1a,b) and containing the same amount of polymerised aluminium species (Al_b_ = 40%) as the PAXXL1911 coagulant. This is of vital importance in terms of the biological stability of the water supplied to the users. The PAXHP908 coagulant was also the most effective in the removal of U_V254_ from 24.32 to 50.53% (Figure 1c).

The analysis of the obtained test results also showed that regardless of the type of coagulant tested in the range of applied doses of tested coagulants DOC fractions characterised by a high content of aromatic rings (UV_254_—Figure 1c) were most effectively eliminated, means that aromatic substances are removed more effectively during coagulation than other organic matter fractions. Absorbance at 254 nm is typical for the aromatic groups with varying degrees of activation. The measurement of absorbance at a wavelength of 254 nm allowed for identifying the DOC fraction containing aromatic rings, and, therefore, characterised by a high potential of forming oxidation and disinfection by-products such as THM [20]. This is very important for the health aspect of water consumers.

**Figure 2:**
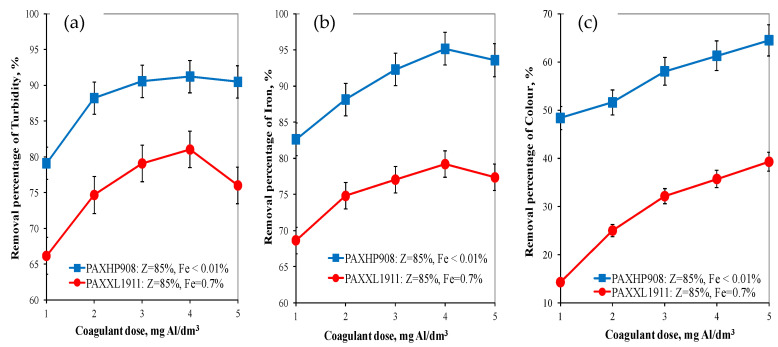
The effect of the type and dose of a coagulant on the efficiency of removing turbidity (**a**), iron (**b**) and colour (**c**).

Analysis of the research results obtained showed that the pH of water after coagulation with the PAXXL1911 coagulant (Z = 85%, Fe = 0.7%) ranged from 7.86 to 8.00 and with the PAXHP908 (Z = 85%, Fe < 0.01%) ranged from 7.85 to 8.00 for doses from 1 to 5 mgAl/dm^3^. The turbidity, colour and concentration of iron was also determined in the water after the coagulation process. Analysis of the obtained test results showed that, as in the case of organic matter removal, the coagulant PAXHP908, with an alkalinity of 85% and no iron in its composition, was the more effective in removing turbidity (from 79.11 to 90.50%), colour (from 48.40 to 64.50%) and iron (from 82.63 to 95.15%) than coagulant PAXXL1911 characterised by an alkalinity of 85% and containing iron compounds in its composition (turbidity: from 66.15 to 80.00%, colour: from 14.30 to 39.30, iron: from 68.65 to 79.20%) (Figure 2a–c).

**Figure 3:**
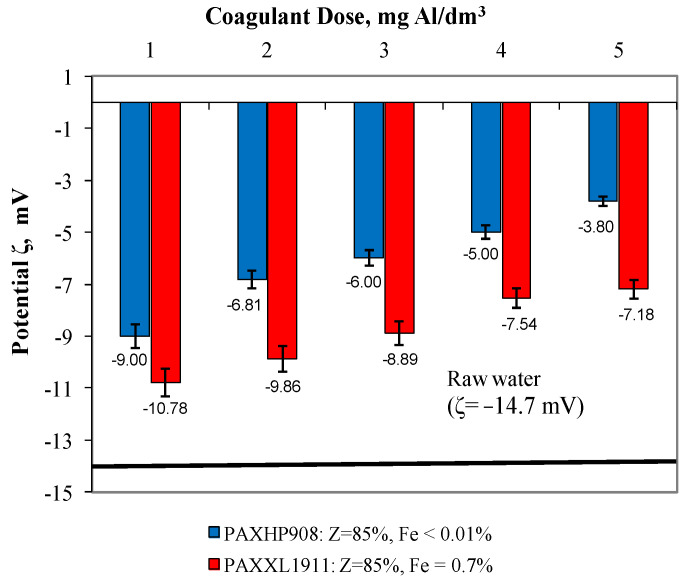
The effect of the type and dose of a coagulant on the change in zeta potential.

In water after coagulation with the less effective coagulant PAXXL1911, the concentrations of iron, colour, total organic carbon and turbidity were, respectively, from 0.223 to 0.310 ± 0.001 mgFe/dm^3^, from 24 to 17 ± 1 mgPt/dm^3^, from 4.954 to 6.627 ± 0.001 mgC/dm^3^ and turbidity from 2.00 to 3.20 ± 0.1 NTU. The effectiveness of the coagulation process with the tested coagulants was also evaluated by measuring the electro-kinetic potential ζ (Figure 3), which determines the stability of the colloids.

According to reports in the literature [25,26,27] with the increase in the absolute value of the potential ζ, the stability of colloids increases. The coagulation process is mainly based on double layer repulsive potential reduction by neutralisation of surface charge of pollutants, and zeta potential is the parameter that provides the information about the charge neutralisation status [28]. In the presented study, we have shown the results of the zeta potential measurement evaluation as a coagulation process control. The extent to which the colloids in the water being treated are destabilised makes a notable contribution to the efficiency of their removal by coagulation. As was expected, the extent of destabilisation increased with the increase in the coagulant dose (which varied from 1 to 5 mgAl/dm^3^). Analysis of the dependences presented in Figure 3 showed that the higher degree of destabilisation of the electro-kinetic potential, which varied between −9.00 mV and −3.80 ± 0.01 mV, was obtained in samples of water during coagulation with polyaluminium chloride PAXHP908 over the entire range of tested doses from 1 to 5 mg Al/dm^3^. The degree of destabilisation of the electro-kinetic potential value after coagulation with coagulant PAXXL1911, characterised by an alkalinity of 85% and containing iron compounds in its composition, ranged from −10.78 mV to −7.18 ± 0.01 mV. The zeta potential for post-coagulation water samples was lower than that found for raw water (−14.7 mV); however, no transition of this potential through the isoelectric point was detected. This may point to the possibility of increasing coagulation effectiveness by an increase in coagulant dosage, but this is not economically justifiable.

**Figure 4:**
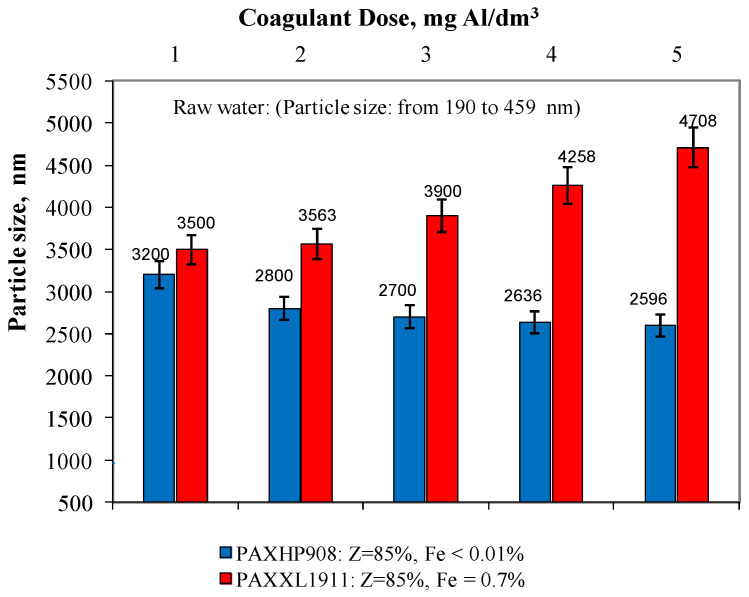
The effect of the type and dose of a coagulant on the change in particle size distribution.

In the water samples after the coagulation process, the particle size was also measured using the dynamic light scattering process (Figure 4). Analysis of the obtained test results showed that in the water after the coagulation of PAXHP908 (Z = 85%, Fe < 0.01%), there were particles whose diameter ranged from 3200 to 2596 ± 1 nm for doses from 1 to 5 mg Al/dm^3^. In water after coagulation with PAXXL1911 (Z = 85%, Fe = 0.7%), the particle diameter was in the range from 3500 to 4708 ± 1 nm. Only in the water samples after coagulation with the coagulant PAXXL1911 characterised by an alkalinity of 85% and containing iron compounds in its composition did the size of the particles remaining in the treated water increase with increasing dosage from 1 to 5 mgAl/dm^3^, and thus with an increase in the amount of iron introduced with the coagulant (Figure 4). Rahman and colleagues [29] proved that iron usually connects with carboxyl groups of organic substances and that iron’s organic complex compound is large and polydispersive with a size of hundreds of nanometres.

Relationship between iron (III), turbidity, colour and organic substances indices, measured as: TOC, DOC and UV_254_ showed strong correlations only in water following coagulation with PAXXL1911 containing iron compounds in its composition (Table 1).

This confirms that coloured iron–organic complexes were created in the water during coagulation PAXXL1911. After the coagulation process with PAXXL1911 polyaluminium chloride, the high Pearson coefficient values were obtained for linear correlations between iron (III) and TOC and between iron (III) and UV_254_ (Table 1), which may indicate that the iron (III) in water formed complexes with organic substances, including dissolved organic substances containing aromatic rings in their composition. The coloured iron–organic complexes formed during PAXXL1911 coagulation most likely exhibited the character of protective colloids, hence leading to the significantly higher turbidity that followed PAXXL1911 coagulation as compared to the turbidity after PAXHP908 coagulation which were, respectively, from 2.00 to 3.20 ± 0.1 NTU (PAXXL1911) and from 1.08 to 1.45 ± 0.1 NTU (PAXHP908). The protective colloids of a hydrophilic nature, are created as a result of the adsorption of organic substances on the surface of iron (III) hydroxide. The organic stabilisation of iron colloids results from the formation of an external encasement containing ionised carboxyl groups [27].

According to numerous researchers [5,25,29,30], one of the reasons for iron stabilisation by organic substances in water may be formation of the so called protective colloids of hydrophilic character. During the research conducted by Albertkiene [30] with the use of groundwater containing iron–organic complexes, it was shown that water pH has the greatest impact on the removal of iron–organic complexes from drinking water during coagulation. Iron–organic complexes are best eliminated from drinking water when its pH is from 6.8 to 6.5 ± 0.1; thus, a rise in pH reduces the effectiveness of their removal. Analysis of the research results obtained showed that the pH of water during coagulation with the PAXXL1911 coagulant (Z = 85%, Fe = 0.7%) ranged from 7.86 to 8.00 ± 0.1 and with the PAXHP908 (Z = 85%, Fe < 0.01%) ranged from 7.85 to 8.00 ± 0.1 for doses from 1 to 5 mgAl/dm^3^. At these pH values, the functional groups of –COOH and –OH organic substances are more reactive in relation to iron ions and they can form iron–organic complexes [27,30,31,32,33,34,35,36].

## 3. Materials and Methods

### 3.1. Water Samples

The subject of the study was a mixture of surface water and groundwater. The groundwater after aeration in forced airflow cascades was mixed with surface water at a volume ratio of 1:3. Raw water was characterised by an increased total iron content from 0.976 to 1.013 ± 0.100 mgFe/dm^3^, iron (II) from 0.136 to 0.139 ± 0.100 mgFe/dm^3^ and iron (III) from 0.840 to 0.874 ± 0.100 mgFe/dm^3^, and increased turbidity from 11.0 to12.3 ± 0.1 NTU, with the intensity of colour from 29 to 31 ± 1 mgPt/dm^3^, a pH of 8.10 ± 0.1 and electro-kinetic potential (ζ) − 14.70 ± 0.01 mV. TOC reached values from 8.048 to 8.215 ± 0.001 mgC/dm^3^, DOC from 7.085 to 7.143 ± 0.001 mgC/dm^3^ and UV_254_ absorption from 19.000 to 19.157 ± 0.001 m^−1^, which indicates that organic substances containing aromatic rings, which are characterised by a high potential of forming oxidation or disinfection by-products, were present among the dissolved substances in the purified water. In raw water the particle diameter was in the range of 190 to 459 ± 1 nm.

### 3.2. Jar Test

Jar tests were carried out by using a 1 L six-place paddle stirrer (Flocculator Kemira 2000, Helsingborg, Sweden). Coagulation was carried out in water samples of 1 L through 1 min fast mixing at a speed of 250 rpm and 25 min flocculation with an intensity of mixing of 30 rpm. The coagulants used were prehydrolysed polyaluminium chlorides with the trade names PAXHP908 and PAXXL1911 that had an alkalinity of 85%, different iron contents (<0.01% and 0.7%) and the same content of polymerised aluminium forms (Al_b_ = 40%). The Al species distribution in the PACls (PAXXL1911 and PAXHP908) samples were analysed by using Ferron complexation timed spectrophotometry. The doses of coagulants were expressed in mg Al/dm^3^ and varied from 1 to 5 mg Al/dm^3^. After coagulation, the samples were subject to sedimentation process for 1 h. The jar tests were repeated three times and the presented results are the average values.

### 3.3. Analytical Methods

The physical-chemical composition of both the raw water and treated water was determined according to the International Standard methods. The organic substances concentration was monitored by measuring the TOC, DOC and UV absorbance at 254 nm. The TOC and DOC were measured using the thermal method and a Shimadzu TOC analyser (Shimadzu Corporation, Kyoto, Japan). DOC was analysed by the TOC analyser after filtration through 0.45µm pore diameter membranes. UV absorbance at 254 nm (UV_254_) was measured by a UV-VIS spectrophotometer Agilent Cary 60 (Agilent Technologies, Inc. Santa Clara, CA, USA) using a quartz cell with a 1 cm path length after filtration through a 0.45µm membrane. The colour (according to Pt scale), total iron and iron (II) concentrations were determined with the Dr 3900 (HACH Lange, CO, USA) spectrophotometer. Iron (II) was measured using the 1,10 phenanthroline method. Total iron was measured using the same method. As a reducing agent of ferric ions to the ferrous ions, hydroxylamine hydrochloride was used. The Al species distribution in the PACls (PAXXL1911 and PAXHP908) samples was analysed by Ferron complexation timed spectrophotometry [16] Al^3+^ reacts with Ferron reagent (Sigma-Aldrich, St. Louis, MO, USA) to form an Al-Ferron complex at pH = 5, λ = 370 nm. An Agilent Cary 60 spectrophotometer was used to measure the Al-Ferron kinetics. Based on the kinetic difference of reactions between Ferron reagent (8-hydroxy-7-iodo-5-quinoline sulfonic acid) with different hydrolysed species, hydrolysed Al species can be divided into three types: monomeric Al species (Al_a_) (instantaneous reaction: 0 to 1 min), medium polymerised Al species (Al_b_) (reaction within 120 min) and species of colloidal (Al_c_) (no reaction in 120 min). The results are shown in Table 2. 

The temperature and pH of the raw water and the purified water were determined with an WTW Multi Line P4 with a combination pH electrode with temperature corrections. Turbidity was measured using the Hach 2100N Turbidimeter (Hach Company, Loveland, CO, USA). Measurement of the electro-kinetic potential ζ was made in raw water samples and after the coagulation process using the Zetasizer Nano Z Analyzer (Malvern Panalytical Ltd., Malvern, UK), which calculates the Zeta potential by determining the electrophoretic mobility of the particles using the laser technique of speed measurement based on the Doppler effect. In the water, the particle size was also measured using the Zetasizer Nano Analyzer. The Zetasizer Nano Analyzer measures particle size using the dynamic light scattering (DLS, dynamic light scattering) process, also known as photon correlation spectroscopy (PCS, photon correlation spectroscopy), which measures Brown’s motion and calculates particle size on this basis.

## 4. Conclusions

The effectiveness of purifying water in the coagulation process was determined by the type of tested polyaluminium chloride. The reason behind the lower effectiveness of water purification in the coagulation process upon applying PAXXL1911, characterised by an alkalinity of 85% and containing higher amounts of iron compounds in its composition than the PAXHP908, was the formation of iron–organic complexes, most likely in the form of protective colloids. The reason behind the formation of coloured iron–organic complexes during coagulation with the highly alkaline PAXXL1911 coagulant may have been the high pH of approx. 8, at which the functional groups of organic substances are more reactive in relation to iron, and the introduction of additional iron ions along with the coagulant, which may have reacted with the organic substances present in the purified water, contributing to the formation of additional iron–organic complexes difficult to remove during coagulation. Therefore, highly basic polyaluminium chlorides containing iron admixtures should not be used to treat water containing above-standard concentrations of organic substances.

## Figures and Tables

**Table 1:** Relationship between the iron, colour, turbidity and the organic matter indices of the water samples after PAXXL1911coagulation.

Linear Correlation Equation	Coefficient of the Pearson Correlation (R)	Range of Concentrations
Turbidity = 9.0257Fe(III) + 0.4788	0.9832	2.0–3.2 NTU 0.174–0.305 mgFe/dm^3^
Fe(III) = 0.0461TOC − 0.0332	0.9839	0.174–0.305 mgFe/dm^3^ 4.954–6.627 mgC/dm^3^
Colour = 4.1005DOC − 3.9876	0.9913	16–23 mgPt/dm^3^ 4.940–6.620 mgC/dm^3^
Colour = 1.7946UV_254_ − 4.3651	0.9883	16–23 mgPt/dm^3^ 11.530–15.900 m^−1^
Fe(III) = 0.0206UV_254_ − 0.1334	0.9989	0.174–0.305 mgFe/dm^3^ 11.530–15.900 m^−1^

**Table 2:** Selected properties of the tested coagulants.

Indicator	Type of Coagulant	
	PAXXL1911	PAXHP908
Alkalinity ratio, [OH^−^]/[Al^3+^]	2.55	2.55
Alkalinity, %	85	85
Al^3+^, %	11.5	8.0
Fe_tot_, %	0.7	<0.01
Monomeric Al species (Al_a_), %	14.2	14.3
Polymerised Al species (Al_b_), %	40.0	40.0
Colloidal Al species (Al_c_), %	45.8	45.7

## Data Availability

Not applicable.

## References

[1] Dąbrowska L. (2016). Removal of organic matter from surface water using coagulants with various basicity. J. Ecol. Eng..

[2] Wang W., Yang H., Wang X., Jiang J., Zhu W. (2010). Effects of fulvic acid and humic acid on aluminum speciation in drinking water. J. Environ. Sci..

[3] Krupińska I. (2016). The Influence of Aeration and Type of Coagulant on Effectiveness in Removing Pollutants from Groundwater in the Process of Coagulation. Chem. Biochem. Eng. Q.

[4] Krupińska I. (2017). The impact of potassium manganate (VII) on the effectiveness of coagulation in the removal of iron and manganese from groundwater with an increased content of organic substances. Civ. Environ. Eng. Rep..

[5] Krupińska I. (2016). The impact of the oxidising agent type and coagulant type on the effectiveness of coagulation in the removal of pollutants from underground water with an increased content of organic substances. J. Environ. Eng. Landsc..

[6] Nowacka A., Włodarczyk Makuła M., Macherzyński B. (2014). Comparison of effectiveness of coagulation with aluminum sulfate and pre-hydrolyzed aluminum coagulants. Desalination Water Treat..

[7] Nowacka A., Włodarczyk-Makuła M., Tchórzewska-Cieślak B., Rak J. (2016). The ability to remove the priority PAHs from water during coagulation process including risk assessment. Desalination Water Treat..

[8] Sogaard E. (2002). Production of the coagulation agent PAX-14. Contents of polyaluminium chloride compounds, Chem. Water Wastewater Treat..

[9] Sillanpää M., Ncibi M., Ch Matilainen A., Vepsäläinen M. (2018). Removal of natural organic matter in drinking water treatment by coagulation: A comprehensive review. Chemosphere.

[10] Rusinowski H., Stanek W. (2007). Neural modelling of steam boilers. Energy Convers. Manag..

[11] Azhar A.T.S., Nordin N.S., Azmi M.A.M., Embong Z., Sunar N., Hazreek Z.A.M., Aziman M. (2018). The Physical Behavior of Stabilised Soft Clay by Electrokinetic Stabilisation Technology. J. Phys. Conf. Ser..

[12] Matilainen A., Vepsäläinen M., Sillanpää M. (2010). Natural organic matter removal by coagulation during drinking water treatment: A review. Adv. Colloid Interfac..

[13] Duan J.M., Gregory J. (2003). Coagulation by hydrolysing metal salts. Adv. Colloid. Interfac..

[14] Hussain S., Van Leeuwen J., Chow Ch Beecham S., Kamruzzaman M., Wang D., Drikas M., Aryal R. (2013). Removal of organic contaminants from river and reservoir waters by three different aluminum-based metal salts: Coagulation adsorption and kinetics studies. Chem. Eng. J..

[15] Pernitsky D., Edzwald J. (2006). Selection of alum and polyaluminum coagulants: Principles and applications. J. Water Supply Res..

[16] Zhou W., Gao B., Yue Q., Liu L., Wang Y. (2006). Al-Ferron kinetics and quantitative calculation of Al(III) species in polyaluminum chloride coagulants. Colloid Surf. A.

[17] Sieliechi J.M., Kayem G.J., Sandu I. (2010). Effect of water treatment residuals (aluminium and iron ions) on human health and drinking water distribution systems. Int. J. Conserv. Sci..

[18] Piekutin J., Skoczko I., Ignatowicz K. (2016). Use of integrated process of petroleum removal from water. Desalination Water Treat..

[19] Lin Lin J., Huang Ch Dempsey B., Hu J.G. (2014). Fate of hydrolyzed Al species in humic acid coagulation. Water Res..

[20] Wolska M. (2018). Removal of precursors of chlorinated organic compounds in selected water treatment processes. Desalination Water Treat..

[21] Riedel T., Biester H. (2012). Molecular Fractionation of Dissolved Organic Matter with Metal Salts. Environ. Sci. Technol..

[22] Tang H., Luan Z. (2003). Differences in coagulation efficiencies between PACl and PICl. J. Am. Water Works Assoc..

[23] Wang D., Tang H., Gregory J. (2002). Relative importance of charge neutralization and precipitation on coagulation of Kaolin with PACl: Effect of sulfate ion. Environ. Sci. Technol..

[24] Krupińska I. (2020). Aluminium drinking water treatment residuals and their toxic impact on human health. Molecules.

[25] Krupińska I. (2018). Removal of natural organic matter from groundwater by coagulation using prehydrolysed and non-prehydrolysed coagulants. Desalination Water Treat..

[26] Krupińska I. (2020). The effect of the type of hydrolysis of aluminum coagulants on the effectiveness of organic substances removal from water. Desalination Water Treat..

[27] Krupińska I. (2021). Removing iron and organic substances from water over the course of its treatment with the application of average and highly alkaline polyaluminium chlorides. Molecules.

[28] Singha N.K., Pandeya S., Singh S., Singh S., Kazmi A.A. (2016). Post treatment of UASB effluent by using inorganic coagulants: Role of zeta potential and characterization of solid residue. J. Environ. Chem. Eng..

[29] Rahman M.A., Hasan M.A., Rahim A., Shafigul Alam A.M. (2010). Characterization of humic acid from the river bottom sediments of Burigonga: Complexation studies of metals with humic acid. Pak. J. Anal. Environ. Chem..

[30] Albrektiene R., Rimeika M., Lubyte E. The removal of iron-organic complexes from drinking water using coagulation process [CD]. Proceedings of the 8th International Conference “Environmental Engineering”.

[31] Liu X., JunLu X., Fang X., Zhou J., Chen Q. (2022). Complexation modelling and oxidation mechanism of organic pollutants in cotton pulp black liquor during iron salt precipitation and electrochemical treatment. Chemosphere.

[32] Okoro B.U., Sharifi S., Jesson M.A., Bridgeman J. (2021). Natural organic matter (NOM) and turbidity removal by plant-based coagulants: A review. J. Environ. Chem. Eng..

[33] Dayarathne H.N.P., Angove M.J., Aryal R., Abuel-Naga H., Mainali B. (2021). Removal of natural organic matter from source water: Review on coagulants, dual coagulation, alternative coagulants, and mechanisms. J. Water Process Eng..

[34] Song J., Jin P., Jin X., Wang X.C. (2019). Synergistic effects of various in situ hydrolyzed aluminum species for the removal of humic acid. Water Res..

[35] Yue Y., An G., Liu L., Lin L., Jiao R., Wang D. (2021). Pre-aggregation of Al_13_ in optimizing coagulation for removal of humic acid. Chemosphere.

[36] Mousavi S.M., Hashemi S.A., Gholami A., Omidifar N., Zarei M., Bahrani S., Yousefi K., Chiang W., Babapoor A. (2021). Bioinorganic Synthesis of Polyrhodanine Stabilized Fe_3_O_4_/Graphene Oxide in microbial supernatant media for anticancer and antibacterial applications. Hindawi Bioinorg. Chem. Appl..

